# Perinatal fetal loss grief counselling experience for expectant fathers: a qualitative study

**DOI:** 10.3389/fpsyt.2025.1685109

**Published:** 2025-12-05

**Authors:** Qiong Gao, Lili Zhu, Hua Guo, Zhiling Zhu, Fuqin Zhang, Yiqin Wang, Beijie Hou

**Affiliations:** 1 Delivery Room, Xinxiang Central Hospital, Xinxiang, Henan, China; 2 School of Nursing, Henan Medical University, Xinxiang, Henan, China; 3 Oncology Ward, Xinxiang Key Laboratory for Palliative Care Skills in Clinical Practice, Xinxiang Central Hospital, Xinxiang, Henan, China; 4 Xinxiang Central Hospital Department of Preventive Health Care, Xinxiang, Henan, China

**Keywords:** prospective father, perinatal loss of fetus, bereavement care, qualitative study, obstetrical nursing

## Abstract

**Background:**

Expectant fathers may experience negative emotions such as grief and distress following perinatal fetal death, potentially sustaining psychological trauma of varying severity. In severe cases, this may adversely affect family harmony, marital intimacy, and social cohesion. Consequently, understanding fathers’ experiences within such circumstances is of paramount importance.

**Aims and objectives:**

To explore the experience of grief counselling for expectant fathers following perinatal fetal loss.

**Methods:**

Sixteen expectant fathers who experienced perinatal fetal loss were recruited for semi-structural interviews between January and December 2024 from a tertiary hospital in Xinxiang, Henan. Transcripts of interviews were analyzed using the Colaizzi 7-step analysis method.

**Results:**

Three themes and nine sub-themes were identified, including the need for grief counselling (hidden self-needs and needs corresponding to psychological development stages), the coexistence of negative and positive emotions (pain, sorrow, helplessness, frustration, acceptance, blessings, and prayers), and the desire for support (emotional support, medical support, and positive self-identity).

**Conclusion:**

Nursing staff should accurately assess the grief counselling needs of fathers who have experienced perinatal fetal loss, and provide them with multifaceted support to alleviate negative emotions. It is recommended that healthcare organizations form multidisciplinary professional teams to provide support and assistance to fathers to help them positively cope with negative emotions and future life and to promote the development of perinatal grief counselling.

## 1 Introduction

The term “perinatal fetal loss” describes a range of outcomes, including maternal miscarriage, induced labor, stillbirth, and neonatal death. These events occur between 20 weeks of gestation and one month after birth (1). According to data from the World Health Organization, nearly 2 million stillbirths in late pregnancy occur worldwide every year (2). China, which accounts for approximately one-fifth of the global population, had a stillbirth rate of approximately 4.9 per 1000 births in 2021 (3). This makes China the fourth highest country in the world in terms of the number of stillbirths (4). It is imperative that medical professionals recognize the traumatic nature of fetal loss, not only for the mother but also for the father. A study (5) emphasizes that fatherhood is an ongoing process of biological and psychological bonding established from conception, with the biological foundations of fatherhood being laid before birth rather than only being recognized as such after the child’s arrival. In some cases, this can result in feelings of sadness, distress, and psychological trauma. Such experiences could potentially disrupt familial harmony, intimate husband-wife relationships, and social cohesion (6–8). After experiencing perinatal loss, expectant fathers frequently assume the responsibility of providing support to their spouses and families. This renders them incapable of articulating their emotions, including but not limited to sadness, anger and guilt (9). They may also suffer from disenfranchised grief. Disenfranchised grief refers to the phenomenon where, following perinatal loss, society often regards maternal sorrow as “legitimate” mourning, whilst the father’s grief over the deceased fetus is frequently marginalized. This marginalization prevents open expression of his sorrow or leads to the devaluation of his emotional investment, thereby depriving his grief of legitimacy (10). Meanwhile, society’s gender stereotypes that demand men to be strong, prevent their grief from being fully released, which may further lead them to adopt unhealthy coping mechanisms like avoidance. Grief counseling is a type of talk therapy that offers emotional support and guidance. The program has been developed to assist mothers, fathers, or other family members who have experienced perinatal loss in re-identifying the unique traumatic triggers inherent to this period. The intervention under discussion addresses the stigma associated with perinatal loss, facilitates the expression of bereavement, and supports individuals in navigating their grief through healthy coping mechanisms. This process is ultimately conducive to the restoration of their prior social functionality (11, 12). Rather than focusing on whether fathers “move on” from grief, this study examines how they adapt to grief through enduring attachment, based on the theory of enduring attachment. Using the dual-process model, we employed longitudinal follow-up to capture shifts in the needs of fathers as they oscillate between loss-oriented and recovery-oriented states. Drawing on gender role theory, we analyzed how societal gender expectations influence the expression of grief and the acquisition of support among fathers. In summary, this study uses multi-theoretical integration to explore comprehensively the nature, process and specificity of perinatal paternal grief.

A study (13) shows that the men who have experienced perinatal loss demonstrate enhanced social cognition. A attribute that may enable them to recognize their own emotional needs and seek support, yet this potential is often underutilized due to gaps in available resources (14). In certain regions, professional counsellors are available to provide grief counselling for men (15); however, there is a paucity of practical information regarding the support of partners and the management of one’s own grief. A study (16) in the Australian males who have experienced perinatal loss has indicated that these individuals often feel like “forgotten mourners” and express a desire for more follow-up support from hospitals. The study also suggests the implementation of creative strategies and inclusive language to encourage male participation in grief counselling. In contrast, grief counselling for perinatal fetal loss in China is still in its infancy, with most existing research focusing on the psychological feelings and experiences of the mothers and midwives. A study indicates that mothers who experience perinatal fetal loss may benefit from bereavement counselling, which can assist them in releasing negative emotions, alleviating tension and anxiety, mitigating the distress caused by loss, and reducing the incidence of depression (1).

There is a notable lack of exploration of the grief counselling needs, emotions, and coping styles of prospective fathers of perinatally lost fetuses (17, 18). This study used a descriptive qualitative research approach, utilizing semi-structured interviews with expectant fathers who had undergone grief counselling. This was undertaken to gain insight into their psychological feelings and experiences and to establish a theoretical foundation for future grief counselling for this demographic group in China.

## 2 Methods

### 2.1 Study design

This study employed a phenomenological approach to qualitative research, utilizing purposive and snowball sampling techniques to recruit participants and conducting face-to-face semi-structured interviews to collect data. The sample size was determined based on the principle of saturation of thematic content (19), whereby the interviews were concluded when no new themes emerged from the content analysis. The textual data were subsequently analyzed and distilled using the Colaizzi 7-step analysis method. The final sample included 16 cases. The characteristics of the respondents and perinatal fetal loss are shown in **Tables 1**, **2**.

**Table 1:** Sociodemographic characteristics of the participants.

NO.	Characteristic			
	Age (years)	Educational level	Employment	Religious affiliation
F1	27	College	Office worker	No religion
F2	41	College	Freelancer	No religion
F3	33	Undergrad	Teacher	No religion
F4	25	College	Teacher	No religion
F5	39	Postgrad	Engineer	No religion
F6	33	College	Worker	No religion
F7	22	Secondary	Worker	No religion
F8	32	Postgrad	Scientist	Buddhism
F9	32	Postgrad	Doctor	No religion
F10	26	Undergrad	Psychologist	No religion
F11	33	College	Office worker	Buddhism
F12	32	Postgrad	Teacher	No religion
F13	39	Undergrad	Public servant	No religion
F14	18	Primary school	Farmer	No religion
F15	32	Postgrad	Policeman	No religion
F16	33	Undergrad	Nurse	No religion

**Table 2:** Characteristics of the perinatal fetal lossed.

Sex	Gestational age (weeks)	Gravidity	Parity	Singleton or not	Diagnosis
Male	27 + 5	2	1	Yes	Trisomy 21
Female	28 + 6	2	2	Yes	Widening of the right lateral ventricle
Male	20 + 1	3	1	No	Fetal chromosomal abnormalities
Male	22 + 5	2	1	Yes	Cleft lip and palate
Male	20	4	2	Yes	Small kidney
Male	23	4	1	Yes	Skin defects
Male	23 + 5	1	0	Yes	Tetralogy of Fallot
Male	21 + 1	3	1	Yes	Hydrocephalus
Female	35 + 5	3	1	Yes	Congenital heart disease
Female	26	1	0	Yes	Kodai syndrome
Female	24 + 1	2	1	Yes	Fetal agenesis
Male	33 + 1	3	2	Yes	Right choroid plexus cyst
Female	23 + 5	2	1	Yes	Anencephaly
Male	32 + 1	2	2	Yes	Spina bifida
Female	28	2	2	Yes	Bladder exstrophy
Female	25 + 1	1	0	Yes	Congenital diaphragmatic hernia

A grief counselling team was established, comprising five members with a bachelor’s degree or above. Two postgraduate students qualified as psychological counsellors were responsible for conducting the interviews, providing psychological counselling, and collecting and analyzing the data. Two head nurses with over 20 years of experience in obstetrics and gynecology nursing were responsible for coordinating and supervising the interviews. An obstetrician was responsible for evaluating the psychological well-being of the prospective fathers. All team members underwent training in qualitative research methods.

This study determined the grief counselling process by an extensive review of the extant literature on the subject and after gathering the opinions of relevant experts. The details of these expert opinions are presented in **Table 3**. Similarly, the initial interview outline was formulated through a comprehensive literature review and consultation with obstetric experts. This was followed by pre-interviews with two expectant fathers who had experienced perinatal fetal loss. The content of these interviews was analyzed, the interview outline was refined, and the need for grief counselling was incorporated, leading to the development of the formal interview outline. The interviews encompassed the following: (1) Rationale for grief counselling: reasons for opting in, and the decision-making process. (2) Experiences during grief counselling: physical and emotional responses and changes undergone. (3) Further considerations and suggestions for grief counselling. Finally, we inquired about the necessity for grief counselling.

**Table 3:** The grief counselling process.

Step	Process
	1. At the time of study admission: The investigator was tasked with the responsibility of assessing, educating, and introducing the purpose and significance of the study to the prospective fathers, following the established inclusion criteria. This was followed by the signing of an informed consent form.
Preparation on admission	2. Environment preparation: The researcher was tasked with preparing a quiet, comfortable, and private room, playing soothing music, and placing papers, pens, towels, and other items in the room to help the participants confide in the researcher.
	3. Preparation of the fetus: The researcher transferred the fetus to a suitable location, performed a thorough cleansing of the infant’s body, arranged its facial features in a way that adhered to the established hospital protocols, and dressed it following established guidelines. In instances where the infant exhibited severe facial deformities or when the prospective father expressed a preference to avoid direct interaction with the fetus, a rag doll was employed as a substitute.
	4. Personnel preparation: A physician from the bereavement counselling team should evaluate the physical well-being of the prospective father.
Grief counselling content	5. The guidance provided by the counselling team is of particular interest in this regard. The team is tasked with the responsibility of gently explaining the characteristics of the deceased infant. This is followed by an opportunity for the father to look at or hold the baby and objects related to it to express his feelings. Examples of such objects include bracelets, footprints, and photographs.
	6. It is recommended that the time with the infant be allocated according to the preferences of the guardian.
After grief counselling	7. Postgraduate students who are qualified as psychological counsellors have been assigned to pay timely attention to the anxiety and painful feelings of the prospective fathers, thereby strengthening psychological guidance and encouragement. They are also responsible for handling the baby’s body, following relevant hospital regulations, and keeping records.
Grief counselling process	8. It has been demonstrated that one-to-one targeted counselling can be administered to prospective fathers during the counselling process, should they encounter severe grief reactions that hinder their ability to proceed.

### 2.2 Setting

The study was conducted in the Department of Obstetrics and Gynecology of a tertiary hospital with 3,200 beds in Xinxiang City, Henan Province. Xinxiang City is a modern urban center with a significant migrant population from across China. In 2024, the hospital had three maternity wards with an annual delivery capacity of over 10,000 babies. In the preceding year, 100 mothers had experienced stillbirths. The current study was conducted between January and December 2024.

### 2.3 Participants and recruitment

#### 2.3.1 Inclusion criteria

The inclusion criteria for perinatal fetal loss were as follows: gestational age of ≥20 weeks; neonates who died within one month of delivery; loss of the fetus due to spontaneous abortion, stillbirth, fetal congenital anomalies, accidents, or maternal factors. The inclusion criteria for the prospective fathers of perinatally lost fetuses were as follows: age ≥18 years; voluntary participation in this study; fluency in Chinese, with the ability to comprehend the purpose of the study, articulate views and ideas, and complete the interviews independently.

#### 2.3.2 Exclusion criteria

The exclusion criteria for prospective fathers of perinatally lost fetuses included those with severe hearing, psychiatric, or intellectual impairments who could not complete the interview and those who were unwilling to participate in the research.

### 2.4 Data analysis

Two researchers transcribed the audio-recorded material into a written form within 24 hours of the interview to guarantee the punctuality and precision of the interviews (20). After the textual data had been verified, all the original recordings were permanently deleted. This process was documented and archived by the Institutional Review Board of the research team for retention over a five-year period. The Colaizzi 7-step analysis method was used to analyze and refine the textual data (21). The interview notes, and the transcribed audio-recorded data were carefully read and analyzed. The significant points were identified and coded, and the coded points were pooled. Common features or concepts were searched for, and points with similar or related features were grouped into themes. The perspectives obtained were verified with the respondents. Two researchers conducted repeated analyses, comparisons, and verifications of the interview data and engaged in discussions. Disagreements were resolved by consultation with the team. This process enabled refining the themes pertaining to the grief counselling experience of fathers who experienced perinatal fetal loss in a precise and comprehensive manner. In order to facilitate the expression of authentic emotional and experiential responses from prospective fathers, the sequence and content of the interview questions can be adapted according to the specific circumstances to ensure the collection of comprehensive data while maintaining the integrity of the interview theme. To protect the confidentiality of the interviewees, the interviews conducted for this study were presented anonymously, with numerical identifiers “F” replacing names. The interview content was initially translated by researchers with professional qualifications in English translation and expertise in perinatal psychology. This was then edited to ensure fluency and naturalness, as well as compliance with academic standards. Personal information is stored separately from research data within the ethics archive of the research institution. De-identified research data and temporary audio files are stored on the same password-protected internal hard drive and are transferred to the institution’s long-term data repository upon completion of the study. Informed consent documents are retained in the ethics archive of the research institution for ten years after the research is published.

### 2.5 Reflexivity

Reflexivity is an integral component of qualitative research, given that researchers’ backgrounds, assumptions, and experiences may influence data collection, analysis, and interpretation (22). The research team comprised three qualitative researchers specializing in perinatal mental health and grief studies. Two researchers hold a Master’s degree in Clinical Psychology, have accrued eight years of experience working with bereaved families, and have no children. This background may have initially emphasized clinical perspectives on grief, potentially overlooking non-clinical expressions of adaptation. The remaining three researchers are mid-career professionals with a minimum of five years of qualitative research experience and are parents of young children (with no history of perinatal loss). Their parental status has led to an increased awareness of family system dynamics. However, this heightened awareness also carries the risk of assuming “universal” parental grief experiences, which could result in the marginalization of fathers’ unique perspectives. All team members acknowledged an initial implicit bias, as evidenced by the framing of fathers’ grief as “less visible” or “more reserved” based on extant literature. This bias may have influenced interview question phrasing or coding decisions. In order to address this, the bracketing technique was employed (23), whereby initial assumptions were documented in a shared reflexive journal at the study’s outset and revisited throughout the analysis to challenge or validate them against emerging data. The study was formally overseen by Professor Zhu, an expert in qualitative reflexivity and grief research. Supervision meetings were convened on a biweekly basis for the purpose of conducting reviews. The following three steps were taken in order to ensure the rigor of the research: The interview transcripts and field notes were examined for signs of researcher bias. Coding decisions were made to ensure alignment with the data rather than assumptions. Interpretive themes were identified to identify potential overemphasis on researcher-held perspectives.

Despite the absence of personal experience with perinatal loss among the researchers, the parental status of two team members necessitated the intentional mitigation of potential bias. Firstly, during the data collection process, the interviewers employed open-ended questions in lieu of assumption-based prompts. This approach enabled the participants to define their own experiences. Secondly, in the analysis stage, the approach of triangulation was employed. This entailed the independent coding of each transcript by two researchers. Discrepancies were resolved through team discussion, with the quotes of the participants serving as the primary source of evidence. Throughout the course of the study, the subjects were required to engage in regular, reflexive journaling. The focus of these entries was on the potential influence of personal contexts on interpretation. Thirdly, the documentation and active addressing of these factors was undertaken in order to minimize the impact of researcher bias, with the aim of ensuring that the findings reflected the participants’ lived experiences rather than preconceived notions. This reflexive process enhances the study’s trustworthiness and transparency (24).

### 2.6 Qualitative rigor criteria

To ensure the credibility and rigor of this study, targeted safeguarding strategies were incorporated into the research design. These strategies were based on the four core standards of trustworthiness in qualitative research (24): credibility, transferability, confirmability and reliability. Regarding credibility, participant verification, sustained engagement, ongoing observation and peer review were employed to ensure that the research findings aligned with the participants’ actual experiences. To ensure transferability, detailed descriptions of the research context, participant characteristics and interview settings were provided, alongside explicit inclusion and exclusion criteria. This allows readers to assess the applicability of the findings to similar contexts or populations. Verifiability was safeguarded by establishing a comprehensive research audit trail and researcher reflection logs. This guarantees that the findings derive from the data, rather than from subjective conjecture. Reliability was ensured through inter-coder reliability testing and standardized data collection procedures. This maintains the consistency of the research outcomes across different researchers and temporal dimensions.

### 2.7 Ethical approval and consent

The study was approved by the Ethics Committee of REDACTED [reference number: 2024-175-01(K)]. The study used a phenomenological approach in qualitative research (25), conducting semi-structured interviews with 16 prospective fathers. The purpose, methodology, and view of the study were explained to the prospective participants before the interview. They were assured that the information disclosed during the interview would be kept strictly confidential. Following the consent to participate, a time was scheduled for the face-to-face interview. Audio recordings were done by a mobile phone, and the participants’ facial expressions, tones of voice, movements, and moods were noted. The participants were at liberty to decline to answer any questions and to withdraw from the study at any time. The sequence and content of the questions were adapted to the circumstances of each interview while ensuring a consistent interview focus to enable prospective fathers to express their authentic feelings and experiences as fully as possible. The interviews were presented in an anonymized format, with numerical identifiers in lieu of names, to safeguard the anonymity of the participants.

## 3 Results

**Figure 1:**
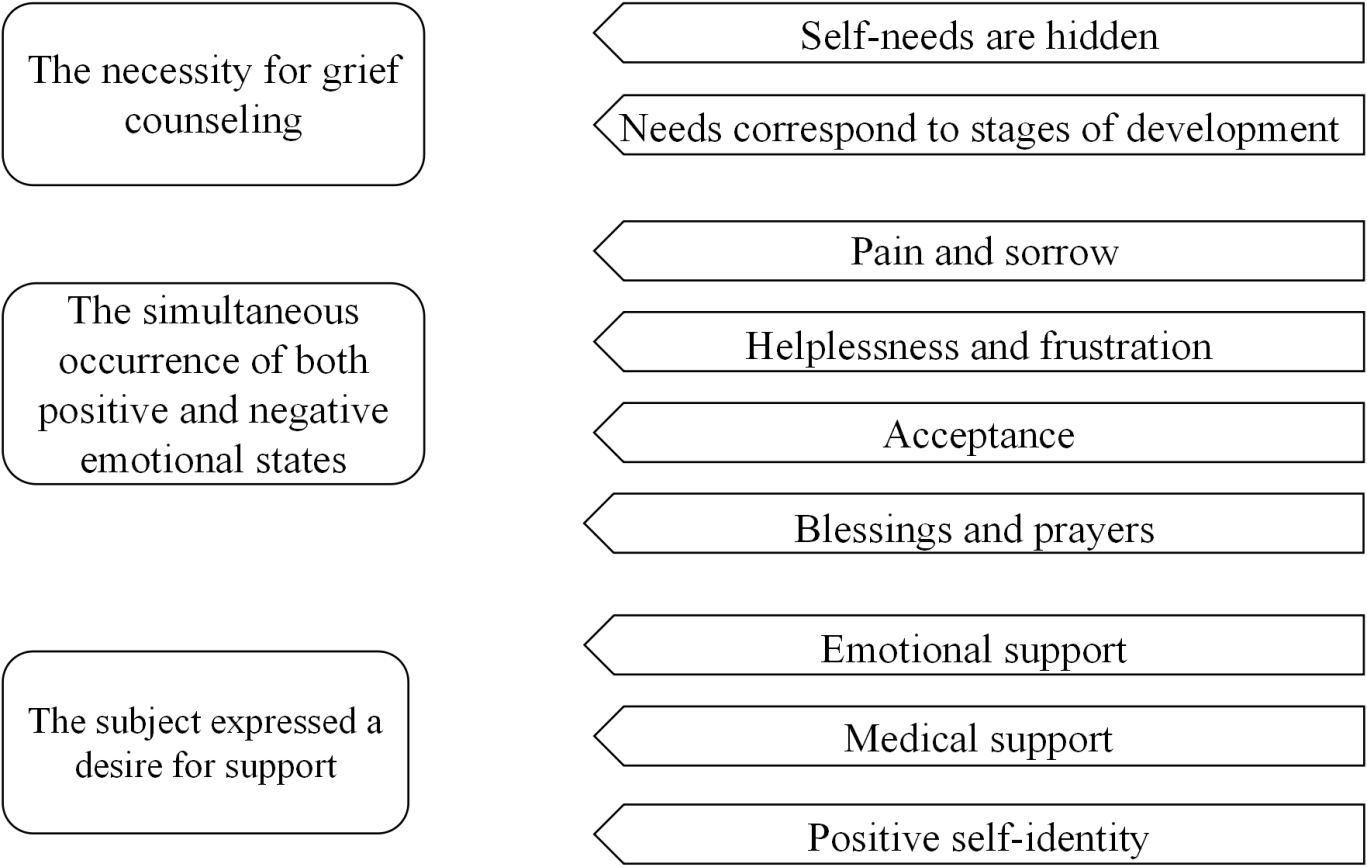
Overview of paternal grief distillation following perinatal fetal loss.

This study distilled three themes: the need for grief counselling, the co-existence of negative and positive emotions, and the desire for support of prospective fathers of perinatally lost fetuses. These themes are detailed in **Figure 1**.

### 3.1 The need for grief counselling

#### 3.1.1 Hidden self-needs

Following the diagnosis of a fetal abnormality, the fathers might experience a range of adverse emotions, including distress and anxiety. To cope with the situation and support his partner and family, he might repress and hide his emotion needs. This can result in the development of a mentality characterized by self-denial, self-reproach, and guilt. While the fathers prioritize caring for his family members, he might lack sufficient self-care. The burden of responsibility and the accompanying feelings of guilt can act as a barrier, preventing individuals from fully acknowledging their true needs.

*“Upon the initial diagnosis of fetal deformity, I experienced a profound sense of despair. However, I was compelled to maintain composure. If I succumbed to emotional distress, it would have a detrimental impact on my wife, who experienced a greater degree of distress than I did. I was compelled to maintain a sense of composure for her benefit.”*

(F2, no religion)

*“As a man, I could not ensure the protection of my children and wife. Consequently, I question my suitability for the roles of a father and a husband.”*

(F1, no religion)

#### 3.1.2 Needs corresponding to stages of psychological development

Upon initial diagnosis of a deformity, the prospective father could not accept the reality of the situation and did not seek professional grief counselling to mitigate his distress and negative emotions.

*“The results of all the preceding tests were within the normal range, yet, presently, I cannot process this information. Furthermore, I am reluctant to engage in grief counselling at this time.”*

(F7, no religion)

*“As a counsellor, I am confident in my ability to navigate this experience independently.”*

(F9, no religion)

Influenced by traditional funeral culture in China, where the child’s accessories are buried with the child, the family is reluctant to accept advice related to grief counselling.

*“Given our decision to relinquish the child, we have no desire to retain any physical reminders of it, including photographs, bracelets, footprints, or other items. Therefore, we propose focusing solely on the child itself.”*

(F8, no religion)

*“The child discloses the truth and will be reincarnated in a new form.”*

(F11, no religion)

*“My mother posited that the infant was underdeveloped and appeared to exhibit a lack of fear, which led her to conclude that it would be better off staying in a different environment.”*

(F10, no religion)

Expectant fathers tend to become more composed over time and acknowledge that they no longer attempt to suppress their emotions. They will then proceed to expose their feelings.

*“If it is not inconvenient, may I request that you allow me one final opportunity to observe you? I wish to express my love for you. I wish you a pleasant journey.”*

(F9, no religion)

### 3.2 The simultaneous occurrence of positive and negative emotional states

#### 3.2.1 Pain and sorrow

The fathers occupy the multifaceted role of a child, a husband, and a child of the parents. Consequently, when the woman in labor experiences profound sadness, the fathers are likely to experience a similar emotional response.

*“Upon observing the evidence of physical abuse on the infant’s body, I could not reconcile the reality of what I was seeing with my initial assumptions. The fetal heart was audible on the previous day.”*

(F1, no religion)

*“We attempted to conceive for a decade before this successful pregnancy of twins. Despite the lengthy bed rest endured by my daughter-in-law, who was at an advanced maternal age, the pregnancy resulted in the loss of the twins.”*

(F3, no religion)

*“This is the second instance a male infant with renal dysfunction is born. The underlying cause of this phenomenon and the reason for its recurrence remain unknown.”*

(F5, no religion)

#### 3.2.2 Helplessness and frustration

A malformation diagnosis is often met with considerable difficulty in accepting by both spouses. The expectant father is frequently uncertain as to the appropriate course of action if induction of labor is necessary because the fetus cannot be saved. Despite their best efforts and the hope of saving the child, they are forced to witness their wife’s distress and the demise of the fetus, which intensifies their feelings of powerlessness and frustration.

*“Non-invasive parental testing for Down Syndrome, using cell-free fetal DNA in the maternal blood, is done for babies who are at high risk. I sought the advice of my friends to ascertain whether I might have a baby like this. However, they could not provide me with the information I sought, and higher-level hospitals could not guarantee the safety of the baby.”*

(F8, no religion)

*“My wife experienced discomfort for 18 hours following the induction of labor. The birth of the infant occurred only after the cervical os had dilated sufficiently. I was motivated to empathize with her pain.”*

(F6, no religion)

*“As a medical practitioner, I am confronted with the daily challenge of diagnosing and treating patients. In this case, the child’s parents have informed me that the infant was born with cardiac issues. It is a dilemma that I must navigate, weighing the potential benefits and risks of various treatment options. In such a situation, the role of the physician is to provide guidance and support, yet the ultimate decision lies with the patient and their family.”*

(F9, no religion)

#### 3.2.3 Acceptance

The role identity of the father undergoes a significant transformation during pregnancy, labor, and the postpartum period. The father assumes the maternal role during pregnancy; however, as the pregnancy progresses and labor is induced, he begins to transition back to his original role as a father. This role change process and adaptation allow the father to accept the new circumstances, adapt to a new identity, and assume the related responsibilities.

*“I request that you allow me some time to interact with the infant in a one-on-one setting. Although I didn’t conceive him., I have been responsible for reading him stories daily since I became aware of his existence.”*

(F15, no religion)

*“I am the third generation of my family, and my relatives consistently expressed a desire for a male child. They encouraged me to observe the child at the end of the day.”*

(F14, no religion)

*“I desired to see the infant in person. Even though he was not born into our family in optimal health, I am grateful that he was born and has become a father figure and a source of stability in my life.”*

(F10, no religion)

#### 3.2.4 Blessings and prayers

Although the father cannot physically participate in the birthing process, he can still express his sentiments by inscribing a card with heartfelt words or offering a personal blessing to the unborn child. This act of love and hope serves as a source of comfort and support during the grieving process.

*“As a Buddhist, I am curious as to whether reciting a scripture to my child would facilitate their reincarnation.”*

(F8, Buddhism)

*“I shall take my child’s bracelet, bury it beneath a tree, and observe its growth and development.”*

(F11, no religion)

*“A small quantity of the infant’s hair is needed, followed by a visit to the temple to offer it a long-life tablet, hoping that the infant will be reincarnated into a suitable family environment in the near future.”*

(F12, no religion)

### 3.3 The subject of an expressed desire for support

#### 3.3.1 Emotional support

The experience of perinatal fetal loss enables fathers to empathize with and understand their wives. They can also perceive the emergence of negative and undesirable emotions, such as sadness and helplessness, in their mothers when they seek emotional support from the wider community, family, and others.

*“It is my sincere hope that the remaining members of the family can empathize with the profound grief and anguish caused by the loss of our child.”*

(F2, no religion)

*“Upon learning that she had to undergo an induced abortion, my wife initially exhibited a loss of temper, resulting in the destruction of various items. Subsequently, she became silent. The atmosphere was palpably dispiriting, and I found myself longing for an opportunity to engage in discourse with others so that they might comprehend the depth of my emotional distress.”*

(F4, no religion)

*“It was a challenging experience for me to be in the ward and observe other families with newborns engaging in joyful and cheerful interactions.”*

(F10, no religion)

#### 3.3.2 Medical support

Expectant fathers reported that grief counselling provided a means of facilitating an early transition from a state of sadness associated with perinatal fetal loss. This process allowed both parents to move on with dignity and few regrets.

*“My wife was in labor with a midwife who was present to guide the labor, provide comfort and reassurance, administer touch and suction to the infant, offer massage, and make sure we felt respected.”*

(F14, no religion)

*“The counselling team provided invaluable support and guidance throughout my experience, offering comfort and sharing insights on the potential outcomes of giving birth in a similar situation. This helped to alleviate feelings of isolation and helplessness.”*

(F12, no religion)

*“Observing my child attired in garments crafted by her grandmother evoked a profound sense of dignity.”*

(F13, no religion)

*“Yesterday, towards the conclusion of the grief counselling session, the nurse extended a gesture of physical affection that conveyed a sense of warmth, understanding, and redemption.”*

(F5, no religion)

#### 3.3.3 Positive self-identity

Grief counselling enables fathers to enhance their positive self-identity and recognize the significance of their personal values and roles, thereby facilitating their active support of their spouses in their physical and emotional recuperation (26).

*“In light of my eldest child’s preparations for the General Certificate of Secondary Education (GCSE) examinations and my elderly mother’s ongoing hospitalization and grief counselling, I was compelled to recognize the necessity of maintaining composure and resilience when the situation demanded it.”*

(F16, no religion)

*“The experience of undergoing grief counselling led to the realization that, although it was not possible to save my child, there were other individuals who could be helped through my role as a police officer.”*

(F15, no religion)

*“I am the sole individual capable of processing this data on behalf of the company. No one else can do so. I must take steps to improve my organization and time management.”*

(F5, no religion)

## 4 Discussion

### 4.1 The necessity for bereavement counselling and psychological support for expectant fathers in the event of perinatal fetal loss is emphasized

The findings of this study (27) indicate that the grief counselling needed by fathers of perinatally lost fetuses is often overlooked or disregarded. This may be because most individuals will prioritize the care of the mother after a traumatic event of perinatal fetal loss, while the psychological feelings of fathers are frequently overlooked and marginalized. Additionally, prospective fathers often choose to conceal their feelings, which is compounded by the perceived inequality in the treatment of natural and induced births by the medical staff. This might result in repressing their feelings and a fear of expressing their genuine needs (11). Furthermore, the perceived unfair treatment of normal and induced labor by medical staff has been found to result in the suppression of feelings and the avoidance of expressing inner needs, a finding that aligns with the observations of (28). This study revealed that the grief counselling needs of prospective fathers undergo dynamic changes at different stages of their psychological development (29). It seems plausible to suggest that this is due to the influence of traditional funeral culture, which might prompt fathers to downplay and ignore their feelings when they first receive the news and suppress their need for grief counselling. However, in the face of the inevitable outcome, fathers might seek positive ways to cope with the situation and choose to receive grief counselling. Research (30) indicates that fathers experience a peak in their need for psychological support 2–4 weeks later than mothers, as they initially focus more on “supporting their partner” rather than processing their own grief. Therefore, healthcare professionals should observe subtle signs of grief in fathers through three approaches: behavioral monitoring, concise and precise questioning, and collaborative identification with partners. This enables the timely detection of psychological shifts at different stages and prioritizes the needs of the expectant father while mediating relationships between him and his family. Care must be taken to prevent funeral customs, personal beliefs or unfounded assumptions from influencing how he expresses his emotions. Following the mother’s hospital admission, healthcare professionals should also provide grief counselling through humanistic care and emotional guidance. This involves encouraging patients to express negative emotions, alleviating internal pressure and reducing the stigma associated with illness. It also enhances patients’ confidence in reintegrating into society and facing the future.

### 4.2 Expectant fathers who have experienced perinatal fetal loss demonstrate a positive response to adverse emotional changes

The findings of this study indicate that expectant fathers experience varying degrees of emotional distress following perinatal fetal loss. This distress can include profound grief and feelings of helplessness. They subsequently come to terms with their situation by offering blessings and prayers for the fetus to be reborn and attain eternal rest in the afterlife of their faith. A study demonstrated that fathers experience varying degrees of anxiety, depression, and post-traumatic stress disorder after experiencing perinatal fetal loss (28). Longer waiting times, diagnostic processes, and treatment durations in hospital settings are associated with more severe maternal anxiety—and this maternal anxiety, in turn, influences fathers’ mood (9, 31). Our study observed that expectant fathers often assume a supportive role, which might result in delayed grief. This can lead to the emergence of inner shame, blame, and guilt, which can further complicate the grieving process (16). Therefore, it is essential to monitor the psychological changes experienced by fathers following perinatal fetal loss. It is crucial for nursing staff to be aware of the potential reasons for these changes and offer grief counselling and support, which can assist fathers in processing their emotions, managing psychological distress, accepting the reality of their loss, and returning to a state of normal functioning.

### 4.3 The objective is to provide multifaceted support for fathers who have experienced perinatal fetal loss to enhance their coping capacity

The findings of this study indicate that expectant fathers who have experienced perinatal fetal loss need emotional support, in addition to assistance from healthcare professionals to enhance their current state and navigate the grieving process. These findings are consistent with the results of an Australian study (27), which indicated that higher levels of support from friends, society, and healthcare professionals were associated with reduced grief levels among fathers. Furthermore, expectant fathers want healthcare providers to be empathetic and caring, to listen to their concerns and to gain an in-depth understanding of their various doubts, worries and anxieties about their unborn child, in order to help them find meaning again (32). Additionally, good communication with mothers was associated with more support for fathers during a loss event, increased self-identity. Therefore, it is recommended that medical and nursing staff utilize the WeChat platform, the online Nail platform, and the offline nursing specialist clinic to implement an “Internet +” type of continuous care service model (33). This would facilitate continued communication between spouses regarding their grief counselling needs and service content. It would be beneficial to encourage couples to open up during labor and postpartum, comfort each other, and work together to overcome their grief and difficulties. They should also strive to understand and affirm the emotional needs of the father at this time (34–36), stimulate the father’s sense of responsibility and purpose, and promote his self-identification with his fatherly role. This will enable him to take measures to recover and restore the harmony of the family.

### 4.4 Theoretical implications and academic contributions

#### 4.4.1 Extending the continuing bonds theory to perinatal fathers’ grief

The finding that fathers primarily engage in “action-oriented bonding behaviors” extends CBT’s application to the unique context of perinatal loss. Traditional CBT research, mostly focusing on spousal or adult bereavement, emphasizes verbalized emotional bonds (37). However, this study reveals that perinatal fathers – shaped by gender role expectations of “strength and action” – construct emotional connections through non-verbal, action-based practices. This not only confirms CBT’s core proposition that “grief is integration of bonds rather than separation” but also adds a new dimension of “action-oriented bonding” to CBT, expanding the theory’s applicability to the understudied group of perinatal fathers.

#### 4.4.2 Cross-validation of the dual process model and gender role theory

The staged grief needs of fathers align with DPM’s dynamic interplay of “loss-oriented” and “restoration-oriented” coping (38), while GRT explains the gender-specific manifestation of this dynamic process. Specifically, fathers’ early focus on emotional validation reflects their unmet need for emotional recognition, which is often suppressed due to societal expectations of “male stoicism” (39, 40). Their mid-term shift to practical tools and long-term focus on meaning reconstruction (balance of two orientations) is a gendered adaptation strategy—fathers channel grief into actionable behaviors to conform to role expectations, rather than engaging in verbal emotional disclosure.

#### 4.4.3 Responding to theoretical controversies in perinatal grief research

This study contributes to resolving the long-standing debate between “grief as recovery” and “grief as continuing bonds” (41) in perinatal loss research. The results show that fathers do not “recover from grief” by forgetting the fetus; instead, they integrate the fetal bond into their identity and daily lives, which is consistent with CBT and contradicts traditional grief work theory. Moreover, the positive association between continuous bonding and adaptive outcomes provides empirical evidence for the superiority of “grief as integration” over “grief as recovery” in perinatal contexts.

## 5 Limitations

The present study is not without limitations. First, although the participants were drawn from a tertiary care hospital, they represented a specific group. Second, the study focused on the internal needs and emotional changes of fathers with perinatal fetal loss. Future research could explore the influencing factors and ways of coping with such situations. The present study also highlighted the ongoing controversy and limited acceptance of grief counselling for fathers, underscoring the necessity for further research in this area. Strengthening publicity through novel media platforms, such as the hospital’s public and video channels, is recommended to enhance public acceptance and recognition. Additionally, the study calls for the development of enhanced service models in hospitals to provide grief counselling, aiming to improve the quality of care for perinatal fetal loss.

## 6 Conclusion

The present study employed qualitative interviews with 16 prospective fathers who had experienced perinatal fetal loss. The analysis yielded three key themes: the need for grief counselling, the coexistence of negative and positive emotions, and the desire for support. It is recommended that healthcare professionals identify the grief counselling needs of fathers with perinatal fetal loss at an early stage. This should be followed by efforts to increase their self-identity, reduce their negative emotions, actively guide them to positively face their loss event, and provide grief counselling. In addition, a multidisciplinary grief counselling care approach should be established, and the content of grief counselling should be improved. This will help fathers to undergo grief counselling for perinatal fetal loss in China.

## Data Availability

The raw data supporting the conclusions of this article will be made available by the authors, without undue reservation.
